# Adjuvant chemotherapy with gemcitabine and cisplatin compared to observation after curative intent resection of cholangiocarcinoma and muscle invasive gallbladder carcinoma (ACTICCA-1 trial) - a randomized, multidisciplinary, multinational phase III trial

**DOI:** 10.1186/s12885-015-1498-0

**Published:** 2015-07-31

**Authors:** Alexander Stein, Dirk Arnold, John Bridgewater, David Goldstein, Lars Henrik Jensen, Heinz-Josef Klümpen, Ansgar W. Lohse, Björn Nashan, John Primrose, Silke Schrum, Jenny Shannon, Eik Vettorazzi, Henning Wege

**Affiliations:** 1 University Medical Center Hamburg-Eppendorf, and University Cancer Center Hamburg, Hamburg, Germany; 2 Tumor Biology Center, Clinic for Medical Oncology, Freiburg im Breisgau, Germany; 3 University College London Cancer Institute, London, UK; 4 University of New South Wales, Sydney, Australia; 5 Vejle Hospital, Vejle, Denmark; 6 Academic Medical Center, Amsterdam, The Netherlands; 7 Southampton General Hospital, Southampton, United Kingdom; 8 CTC North GmbH & Co. KG at the University Medical Center Hamburg-Eppendorf, Hamburg, Germany; 9 University of Sydney, Sydney, Australia

**Keywords:** Cholangiocarcinoma, Gallbladder cancer, Biliary tract cancer, Adjuvant chemotherapy, Biobanking, Shared decision-making

## Abstract

**Background:**

Despite complete resection, disease-free survival (DFS) of patients with cholangiocarcinoma (CCA) is less than 65 % after one year and not more than 35 % after three years. For muscle invasive gallbladder carcinoma (GBCA), prognosis is even worse, with an overall survival (OS) of only 30 % after three years. Thus, evaluation of adjuvant chemotherapy in biliary tract cancer in a large randomized trial is warranted.

**Methods/Design:**

ACTICCA-1 is a randomized, multidisciplinary, multinational phase III investigator initiated trial. With respect to data obtained in the ABC-02 trial, we selected the combination of gemcitabine and cisplatin for 24 weeks as investigational treatment. Based on adjuvant trials in pancreatic cancer with comparable postoperative recovery time, inclusion of patients within a maximum interval of 16 weeks between surgery and start of chemotherapy was stipulated. Due to the different prognosis and treatment susceptibility of muscle invasive carcinoma, two separate cohorts (CCA and GBCA) were included to capture the potentially different treatment effects. Randomization is stratified for lymph node status for both cohorts and localization for CCA. The primary endpoint is DFS and secondary endpoints include OS, safety and tolerability of chemotherapy, quality of life, and patterns of disease recurrence. For CCA, adjuvant chemotherapy should increase DFS 24 months post-surgery from 40 to 55 % to be considered relevant. With a power of 80 % and a significance level of 5 %, 271 evaluable study patients have to be followed for 24–28 months to observe 166 events. For GBCA, chemotherapy should increase DFS 24 months post-surgery from 35 to 55 % to be of relevance; thus, 154 evaluable study patients have to be monitored for 24–28 months to observe 90 events. In both cohorts, randomization will be 1:1 with chemotherapy for 24 weeks and imaging every twelve weeks. In 2014, the study was initiated in Germany and in The Netherlands (funded by the Deutsche Krebshilfe, the Dutch Cancer Society, and supported by medac GmbH). Sites in Australia, Denmark, and the United Kingdom (funded by Cancer Research UK) are joining 2015.

**Trial registration:**

The study is registered with ClinicalTrials.gov (NCT02170090) and the European Clinical Trials Database (2012-005078-70). Registration date is 06/18/2014.

**Electronic supplementary material:**

The online version of this article (doi:10.1186/s12885-015-1498-0) contains supplementary material, which is available to authorized users.

## Background

### Epidemiology of biliary tract cancer

The incidence of biliary tract cancer (BTC) varies extremely in different geographical regions. In Western countries, the rate of intrahepatic cholangiocarcinoma (CCA) is low with 0.4 to 1.0 cases per 100,000. The incidence is highest in patients older than 65 years of age. For unknown reasons, incidence and mortality rates are increasing within the last decades in most developed countries. In contrast, hilar and distal CCA demonstrate only minor regional differences with incidence rates ranging between 0.5 and 1.1 per 100,000. A slight male predominance is found in patients with CCA. Cirrhosis, hepatitis B and C, and primary sclerosing cholangitis are well known risk factors [1–4].

The incidence of gallbladder carcinoma (GBCA) is around 2.0 per 100,000 with a median age at the time of diagnosis of 67 years. Gallstones and chronic infections are important risk factors for GBCA [5, 6].

### Outcome after surgical resection

Currently, complete surgical resection represents the only potentially curative treatment option for CCA and GBCA, and is therefore the treatment of choice if the respective tumor is deemed resectable [7]. More than 50 % of patients present with unresectable disease at the time of diagnosis. The prognosis at this stage is dismal, being approximately 3–5 months without intervention [8, 9], and 6–12 months with palliative chemotherapy (CTx) [10]. Even after curative resection, 5-year overall survival (OS) is only 20–40 %. The most relevant prognostic factors after resection are nodal infiltration, resection margins, vascular invasion, and tumor grading [11–18]. Interestingly, retrospective analysis showed only a minor role for margin status (R0 vs. R1) in the prognosis of CCA following resection, as long as complete tumor clearance is achieved with modern resection techniques. A recent study evaluated the results of surgical therapy for intrahepatic CCA, the incidence, and the management of disease during two sequential periods [19]. The 3-year OS was 62 %, whereas the 3-year disease-free survival (DFS) was only 30 %, the median OS was 57.1 months. Furthermore, von der Gaag and colleagues evaluated the long-term outcome of 175 consecutive patients with resected extrahepatic CCA [20]. In this study, the 2-year OS was 50 % and declined to 26 % after five years. In summary, following complete resection of CCA, patients had DFS rates of 48 to 65 % after one year and 23 to 35 % after three years without adjuvant treatment [12, 17, 21]. Patients with a positive nodal status (N1) and/or vascular invasion (V1) at time of resection had an even higher risk of disease recurrence.

For muscle invasive GBCA, prognosis seems to be even worse [22]. Following complete resection, DFS times are about 10–12 months and OS rates are about 55 % after one year and about 30 % after three years [21–24]. A retrospective Dutch registry study evaluating 368 patients with GBCA and curative surgery between 2003 and 2008 confirmed these data (1-year OS 56 %, 3-year OS 26 %) [25].

### Treatment modalities for unresectable biliary tract cancer

Potential treatment modalities for unresectable BTC include CTx, radiotherapy, chemoradiation, photodynamic treatment, and liver transplantation in localized unresectable CCA. Current approaches for systemic, unresectable BTC, either at initial diagnosis or in case of local or distant disease progression after resection, are based on systemic CTx. A previous randomized trial revealed that CTx significantly improved survival and quality of life compared to best supportive care [26]. Several drugs were found to be active in BTC, e.g., fluorouracil, gemcitabine, mitomycin, cisplatin, capecitabine, epirubicin, and oxaliplatin. A pooled analysis of 104 studies evaluating CTx in advanced BTC suggested that gemcitabine combined with cisplatin or oxaliplatin achieves the best response rates; however, without significantly improving survival [27]. The recent randomized phase III ABC 02 trial revealed a median OS of 11.7 months among 204 patients treated with gemcitabine and cisplatin compared to 8.1 months among 206 patients treated with gemcitabine alone (hazard ratio 0.64, 95 % confidence interval 0.52 to 0.80, p < 0.001). In addition, median progression-free survival and tumor control among patients in the gemcitabine/cisplatin-group was significantly increased (8.0 vs. 5.0 months, *p* < 0.001; 1.4 % vs. 71.8 %, *p* = 0.049). Adverse events (AE) were similar in the two groups, with the exception of more frequent neutropenia in the gemcitabine/cisplatin-group, although the number of neutropenia-associated infections was similar in the two groups [10]. Therefore, the combination of gemcitabine and cisplatin is currently regarded as standard of care in metastatic or unresectable BTC [28].

### Adjuvant chemotherapy for biliary tract cancer

Because of high rates of disease recurrence and poor survival rates following surgical resection, postoperative treatment modalities, e.g., CTx, radiotherapy, and chemoradiation, have been considered to improve patient survival after resection of BTC [16]. Randomized data on the efficacy of adjuvant treatment after resection of BTC are scarce. A multicenter randomized trial evaluated the effect of adjuvant CTx with mitomycin C and fluorouracil compared to surgery alone for patients with pancreato-biliary malignancies [21]. In this trial, a non-significant survival benefit was seen for patients with adjuvant CTx following R0 resection for CCA with a DFS at five years of 32.4 % vs. 15.8 % without adjuvant CTx. In addition, a recent single-institution retrospective evaluation found that gemcitabine-based adjuvant CTx after curative intent resection of CCA significantly improved patient survival [15]. Furthermore, a retrospective analysis of the Surveillance, Epidemiology, and End Results database showed a significant benefit for adjuvant radiation therapy [29]. Finally, combined chemoradiation with fluorouracil and mitomycin in 34 patients seemed to be beneficial compared to historical survival data [30].

The GBCA subgroup of a randomized trial evaluating fluorouracil and mitomycin compared to observation alone, showed a significant increase of 8.7 % in the five year DFS rate in favor of adjuvant CTx in the overall GBCA cohort consisting of patients after curative and non-curative surgery [21]. Additionally, adjuvant fluorouracil-based chemoradiation has been used as adjuvant treatment after complete or margin-positive resection [23, 24, 31, 32]. Although an effect of adjuvant chemo(radio)therapy has been suggested for GBCA, randomized data evaluating current CTx regimens are lacking.

A recent systematic review showed a beneficial impact of adjuvant treatment in BTC, particularly in patients with involved lymph nodes or resection margins and distal or hilar CCA [33]. However, in regard of the paucity of randomized data (only evaluating fluorouracil and mitomycin) the derived recommendation in this review is not without controversy; and thus, current guidelines recommend inclusion in clinical trials [7, 34]. In addition to the ACTICCA-1 trial reported in this paper, two studies are currently underway (recruitment closed, final data awaited) to investigate the role of adjuvant CTx in patients with BTC, the French PRODIGE-12 study evaluating Gemcitbaine and Oxaliplatin (NCT01313377) and the British BILCAP study employing capecitabine (NCT02170090).

### Study rationale

Survival after curative intent resection in BTC is poor due to high rates of disease recurrence. Data from clinical trials and retrospective analyses suggest a benefit for adjuvant treatment. Therefore, the evaluation of adjuvant CTx in BTC is of high clinical relevance. With respect to current data obtained in the large randomized phase III ABC 02 trial [10], the combination of gemcitabine and cisplatin for 24 weeks was selected for this clinical trial as experimental treatment added to the current standard of observation alone [34]. The chosen stratification factors are based on currently available data. Intrahepatic vs. hilar and distal CCA are regarded as different entities of BTC [4]. Therefore, stratification for this trial was stipulated to exclude influence of localization. In regard of the different prognosis and the potentially different treatment susceptibility of muscle invasive carcinoma, separate cohorts for CCA and GBCA were included in the trial design to ensure capturing the potentially different treatment effects for both entities. Although lymphadenectomy was only recently added as standard surgical approach, lymph node status seems to be the most important pathological risk factor for recurrence, as demonstrated in several studies [11, 15, 17, 33]. A recent large retrospective analysis in 449 patients with intrahepatic CCA, of whom 248 received lymph node dissection, demonstrated a significant difference in OS of 30 vs. 24 months (N0 vs. N1) [35]. Similarly, lymph node positivity is a strong prognostic factor in GBCA and was accordingly defined as stratification factor for this cohort as well [23, 32, 36].

### Study objectives

The primary objective of this study is to evaluate the efficacy of gemcitabine and cisplatin and observation in terms of DFS compared to observation alone in patients with BTC after complete surgical resection. The primary endpoint of this study is DFS and secondary endpoints include DFS rate 24 months post-surgery (DFSR@24), OS, safety and tolerability of adjuvant CTx, quality of life (QoL), function of biliodigestive anastomosis (in terms of surgical revision, requirement for additional drainage procedures), rate and severity of biliary tract infections, patterns of disease recurrence, and locoregional control.

### Substudy evaluating the shared decision-making process

**Fig. 1:**
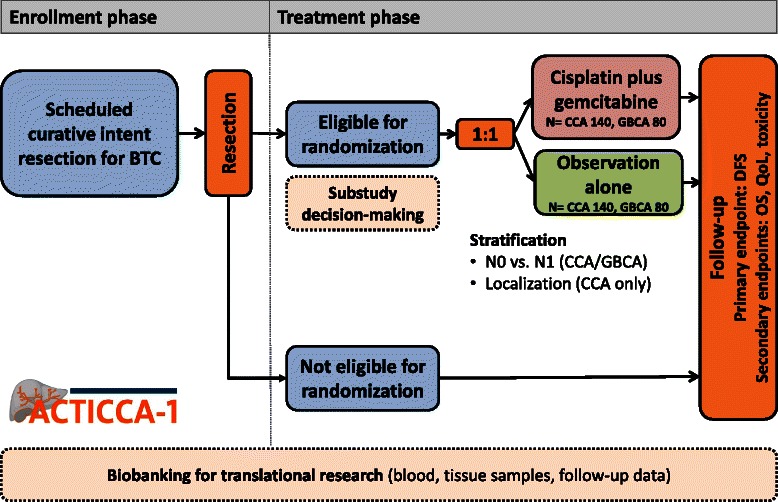
ACTICCA-1 trial design. Legend: BTC, biliary tract cancer; CCA, cholangiocarcinoma; GBCA, gallbladder carcinoma; DFS, disease free survival; OS, overall survival; QoL, quality of life

New media provide fast and easy access to information and enhance patient autonomy. Therefore, patients’ competence concerning the consequences of their disease is growing. Nevertheless, the information and education provided by the physician is not replaced, but becomes even more important. In the doctor-patient relationship, the physician plays a central role as consultant, who not only offers information, but also involves patients in the decision-making process (shared decision-making). Surveys among oncological patients and physicians on the subject of “shared decision-making” show differences between physicians’ and patients’ perspective regarding aims of treatment and involvement in the decision-making process. Therefore, the quantity and quality of information patients have gained after the explanatory discussion and the involvement of patients in the decision-making process will be investigated in a substudy in both cohorts of patients with BTC (Fig. 1) [37, 38].

## Methods/Design

The ACTICCA-1 study is a multinational, prospective, randomized, controlled phase III trial designed to assess the clinical performance of gemcitabine plus cisplatin and observation vs. observation alone in patients after curative intent resection of BTC (Fig. 1). 280 patients (140 patients per arm) in the CCA cohort and 160 patients (80 patients per arm) in the GBCA cohort will be randomized in the treatment phase of the study. Study enrollment will continue until the indicated number of patients is reached. Patients withdrawn from the trial for whatever reason will not be replaced.

### Patient selection and randomization

The study contains two different parts, an enrollment and a treatment phase with different selection criteria. The focus of the enrollment phase is to collect samples for translational research and the corresponding patient data (Fig. 1). Patients will be enrolled into the trial according to the eligibility criteria for the enrollment phase (Table 1) and undergo surgical resection with pre- and postoperative blood sampling and intraoperative tissue sampling. Postoperatively, all enrolled patients are assessed for eligibility for the treatment phase (Table 2). Additionally, patients not previously enrolled into the enrollment phase for whatever reason (e.g. incidental finding of CCA or GBCA during surgery) may enter the treatment phase directly (Fig. 1). These patients have to fulfill the criteria for the enrollment and the treatment phase (Table 1 and Table 2).

**Table 1:** Eligibility criteria for the ACTICCA-1 enrollment phase

• Suspicion of or histologically/cytologically confirmed BTC (intrahepatic, hilar or distal CCA, or muscle invasive GBCA) scheduled for complete surgical resection.
• Written informed consent.
• Age >18 years.
• No prior CTx for BTC.
• No previous malignancy within three years or concomitant malignancy, except non-melanomatous skin cancer or adequately treated *in situ* cervical cancer.
• No severe or uncontrolled cardiovascular disease (congestive heart failure NYHA III or IV, unstable angina pectoris, history of myocardial infarction in the last three months, significant arrhythmia).
• Absence of psychiatric disorder precluding understanding of information of trial related topics and giving informed consent.
• No serious underlying medical conditions (judged by the investigator), that could impair the ability of the patient to participate in the trial.
• Fertile women (<1 year after last menstruation) and procreative men willing and able to use effective means of contraception (oral contraceptives, intrauterine contraceptive device, barrier method of contraception in conjunction with spermicidal jelly or surgically sterile).
• No pregnancy or lactation.

*BTC* biliary tract cancer, *CCA* cholangiocarcinoma, *CTx* chemotherapy, *GBCA* gallbladder carcinoma

**Table 2:** Selection criteria for the ACTICCA-1 treatment phase

• Histologically confirmed BTC (intrahepatic, hilar or distal CCA, or muscle invasive GBCA) after surgical therapy with macroscopically complete resection (mixed tumor entities with hepatocellular carcinoma are excluded).
• Macroscopically complete resection (R0/1) within 6 (−16) weeks before scheduled start of CTx.
• Performance status according to the ECOG of 0–1.
• Adequate hematologic function: ANC ≥1.5 × 10^9^/L, platelets ≥100 × 10^9^/L, hemoglobin ≥9 g/dl or ≥5.59 mmol/L.
• Adequate liver function as measured by serum transaminases (AST and ALT) ≤5 x ULN and bilirubin ≤3 x ULN.
• Adequate renal function, i.e. serum creatinine ≤1.5 x ULN, glomerular filtration rate ≥ 60 mL/min determined with the MDRD formula.
• No active uncontrolled infection, except chronic viral hepatitis under antiviral therapy.
• No concurrent treatment with other experimental drugs or other anti-cancer therapy, treatment in a clinical trial within 30 days prior to randomization.
• Negative serum pregnancy test within 7 days of starting study treatment in pre-menopausal women and women <1 year after the onset of menopause (a negative test has to be reconfirmed by a urine test, should the 7-day window be exceeded).

*ANC* absolute neutrophil count, *BTC* biliary tract cancer, *CCA* cholangiocarcinoma, *CTx* chemotherapy, *ECOG* Eastern Cooperative Oncology Group, *GBCA* gallbladder carcinoma, *MDRD* Modification of Diet and Renal Disease, *ULN* upper limit of normal

After inclusion in the treatment phase, patients will be randomized to arm A or B stratified according to the following criteria:

- Intrahepatic vs. hilar/distal localization for CCA
- Lymph node positivity vs. negativity for CCA and GBCA

### Treatment

Adjuvant CTx is currently not standard of care for BTC. Thus, gemcitabine and cisplatin are defined as investigational medicinal products (IMP) for this study.

#### Arm A (gemcitabine plus cisplatin and observation)

Patients assigned to arm A will be followed every three months and will receive gemcitabine (1000 mg/m^2^) plus cisplatin (25 mg/m^2^) every three weeks on days 1 and 8 intravenously. Treatment with gemcitabine plus cisplatin will be administered until progression or for a maximum of eight three-week cycles (24 weeks). In case of recurrent disease, unacceptable toxicity, or withdrawal of consent, treatment will be terminated.

#### Arm B (standard postoperative management)

Patients assigned to arm B will be followed every three months.

### Assessments during the treatment phase

#### Within four weeks prior to randomization/start of first treatment

- Review of eligibility criteria
- Relevant medical history and demographics
- Obtain surgical and pathological report
- Physical examination and performance status according to Eastern Cooperative Oncology Group (ECOG)
- Laboratory test, including hematology, chemistry panel and CA 19–9
- Serum pregnancy test (for women of child bearing potential)
- Quality of life assessment using the European Organisation for Research and Treatment of Cancer (EORTC) questionnaire QLQ-C30 and the module BIL21
- Audiometry (recommended)
- Documentation of disease status by contrast enhanced abdominal magnetic resonance imaging (MRI) or computed tomography (CT) and chest CT, preoperative imaging can be used if performed within 12 weeks prior to randomization

#### Follow-up (both arms)

All subjects will be followed every three months for two years and afterwards 6-monthly for further three years after randomization. Evaluation for disease recurrence will be performed by clinical visitation including:

- Physical examination
- Laboratory tests and CA 19–9
- QoL assessment using the EORTC questionnaire QLQ-C30 and the module BIL21
- Disease assessment (CT or MRI of chest and abdomen for two years, afterwards abdominal ultrasound), evaluation according to the revised Response Evaluation Criteria in Solid Tumors (RECIST) guideline version 1.1

After disease recurrence, patients will be followed for survival, disease status, and further therapy (via clinical visitation or telephone contact).

### Analyses of study endpoints

The two study cohorts will be analyzed for the primary endpoint (DFS) when 166 events (recurrence or death) in the CCA cohort and 90 events in the GBCA cohort have been observed. Follow-up for OS will continue for up to five years for each individual subject. Time from randomization to date of first observed disease recurrence (either local or distant) or death from any cause (a second malignancy will not be counted as event in the DFS analysis) will be utilized to compare DFS. In order to determine disease recurrence, tumor assessments (contrast enhanced chest CT and CT or MRI of abdomen, serum marker CA 19–9) will be performed every three months for two years and afterwards every six months for further three years by abdominal ultrasound and CA 19–9. In case of clinical suspicion of recurrent disease and/or CA 19–9 elevation without radiological tumor recurrence, further examinations must be performed searching for a local recurrence or metastatic progression of the disease. Diagnosis of recurrence could either be made by radiological imaging or by positive cytology or histology. OS will be determined as time from randomization to date of death.

Safety assessments will include physical examination, performance status (according to ECOG), clinical laboratory values, and concomitant medication. All observed toxicities and side effects will be graded according to NCI CTCAE 4.03 and the degree of association of each event with the intervention will be assessed and summarized. Treatment related serious AE (SAE), defined as SAE considered possibly, probably, or definitely related to treatment, will be determined. QoL will be assessed using the EORTC QLQ C30 questionnaire and the module BIL21 at baseline and every 12 weeks during follow up. Function of biliodigestive anastomosis (in terms of surgical revision, requirement of drainage procedure) will be assessed during the follow up visits. Severity of biliary tract infections will be classified according to NCI CTCAE 4.03. Pattern of recurrence will be classified according to distant vs. local recurrence. Local control will be defined as rate of locoregional failures (local recurrence or locoregional lymph node metastases). Both endpoints will be evaluated with regard to the pathological stage at resection.

### Quality assurance and safety

Patient data are collected in electronic case report forms at the data center of the clinical research organisation (Clinical Trial Center North at the University Medical Center Hamburg-Eppendorf, Hamburg, Germany). Consistency checks will be performed on newly entered forms and queries issued in case of inconsistencies. Monitoring will be performed according to the respective national standards. A data safety monitoring board will review the data on a regular basis.

### Statistical considerations and data handling

All patients receiving at least one dose of study treatment are included in the safety analysis. The intention-to-treat (ITT) population includes all patients in the study (signed consent form and confirmation of eligibility). All patients are grouped according to their randomization, regardless of treatment received. The primary efficacy endpoint is DFS in the ITT population.

For CCA, retrospective analyses after R0/R1 resection showed a DFS@24 of approximately 40 % [12, 15, 17, 19]. Therefore, DFSR@24 is expected to be 40 % without adjuvant treatment. The IMP (adjuvant gemcitabine and cisplatin) should increase DFSR@24 by at least 15 % (to 55 %) corresponding to a hazard ratio of 1.563 to be regarded as promising for further evaluation and of clinical relevance. The risk of falsely rejecting the null hypothesis of no difference between the experimental and the control arm was restricted to 5 %. The risk of falsely rejecting the alternative hypothesis of a difference between the experimental and the control arm was selected not to exceed 20 %, corresponding to a power of 80 %. With these restrictions, 271 evaluable study patients have to be followed for 24–28 months to observe 166 events. With an assumed loss-to-follow-up of 3 %, 280 patients (140 patients per arm) have to be recruited for inclusion into the trial.

For GBCA, retrospective analyses after R0/R1 resection showed a DFSR@24 of approximately 35 % [21–24]. Therefore, DFSR@24 is expected to be 35 % without adjuvant CTx. The IMP should increase DFSR@24 by 20 % (to 55 %) to be regarded as relevant. Employing the same statistical restrictions as for CCA, 154 evaluable study patients have to be followed for 24–28 months to observe 90 events. With an assumed loss-to-follow-up of about 4 %, 160 patients (80 patients per arm) have to be recruited for inclusion into the trial.

Both cohorts are analyzed using the two-sided two-sample log-rank test, following a group-sequential plan according to O’Brien and Fleming. This plan provides one interim analysis after 110 events for the CCA cohort and after 59 events for the GBCA cohort, and one final analysis when 166 events in the CCA cohort and 90 events in the GBCA cohort have occurred. For both cohorts, the interim analyses will have a power of 43.3 % if the assumed hazard ratio of 1.563 in the CCA cohort and 1.854 in the GBCA cohort. In both cohorts, the power increases to 90.4 % if the hazard ratio is 2.

### Ethical aspects, trial registration

The ethics committee of the Ärztekammer Hamburg approved the ACTICCA-1 trial as leading ethics committee for all German sites (PVN4571). In addition, local ethics committees approved the participating German sites (for a complete list see Additional file 1). For The Netherlands, the study was approved by the Medisch Ethische Toetsingscommissie of the Academic Medical Center of the University of Amsterdam. The trial is registered with ClinicalTrials.gov (NCT02170090) and the European Clinical Trials Database (2012-005078-70).

### Funding

The Deutsche Krebshilfe (grant number 110215), the Dutch Cancer Society, and Cancer Research UK currently fund the ACTICCA-1 trial. Other international partners are seeking their own funding sources. For Germany and The Netherlands, gemcitabine and cisplatin are provided by medac GmbH (Wedel, Germany). In the United Kingdom, funding by Cancer Research UK includes the medication.

### Biobanking and translational research

Data on prognostic factors for BTC are rare. Moreover, if adjuvant CTx will become a standard of care in the future, predictive markers might gain particular importance. Within the current trial, tumor tissue and serum (both stored locally) will be collected together with the clinical data. Besides the clinical case report form, an allocation database will be established gathering the data of the available patient samples at each study site to enable translational research. Translational research will be performed to evaluate the prognostic and predictive impact of different blood and tissue markers in BTC with particular regard to adjuvant CTx with gemcitabine plus cisplatin.

## Additional file

Additional file 1:
**List of involved German ethics committees.**

## Abbreviations

AE: Adverse event
BTC: Biliary tract cancer
CCA: Cholangiocarcinoma
CT: Computed tomography
CTx: Chemotherapy
DFS: Disease-free survival
DFS@24: Disease-free survival 24 months after resection
ECOG: Eastern Cooperative Oncology Group
EORTC: European Organization for Research and Treatment of Cancer
GBCA: Gallbladder carcinoma
IMP: Investigational product
ITT: Intention-to-treat
MRI: Magnetic resonance imaging
OS: Overall survival
QoL: Quality of life
RECIST: Response Evaluation Criteria In Solid Tumors
SAE: Serious adverse event
