# Supplementary material for: Adjuvant chemotherapy with gemcitabine and cisplatin compared to observation after curative intent resection of cholangiocarcinoma and muscle invasive gallbladder carcinoma (ACTICCA-1 trial) - a randomized, multidisciplinary, multinational phase III trial
Source: BMC Cancer. 2015 Jul 31;15:564. doi: 10.1186/s12885-015-1498-0 (PMC4520064; doi:10.1186/s12885-015-1498-0)
Supplement: Additional file 1: — List of involved German ethics committees. [file 12885_2015_1498_MOESM1_ESM.pdf]

Liste der Ethik-Kommissionen

Zentrum 01 - Universitätsklinikum Hamburg-Eppendorf

Ethik-Kommission der Ärztekammer Hamburg
Weidestr. 122 b

22083 Hamburg

Zentrum 02 - Universitätsklinikum Aachen

Ethik-Kommission an der Medizinischen Fakultät der RWTH Aachen
Pauwelsstr. 30
52074 Aachen

Zentrum 03 - Charité -Universitätsmedizin Berlin

Ethik-Kommission des Landes Berlin
Fehrbelliner Platz 1
10707 Berlin

Zentrum 05 - Universitätsklinikum Carl Gustav Carus

Ethikkommission der Medizinischen Fakultät Carl Gustav Carus der Technischen Universität Dresden
Fetscherstr. 74
01307 Dresden

Zentrum 07 - Universitätsklinikum Essen

Ethik-Kommission der Medizinischen Fakultät der Universität Duisburg-Essen
Robert-Koch-Str. 9-11
45147 Essen

Zentrum 08 - Klinikum der J.W. Goethe-Universität

Ethik-Kommission des Fachbereichs Medizin der Johann Wolfgang
Goethe-Universität
Haus 1
Theodor-Stern-Kai 7
60590 Frankfurt/Main

Zentrum 09 - Medizinische Hochschule Hannover

Ethikkommission der Medizinischen Hochschule Hannover
Carl-Neuberg-Str. 1
30625 Hannover

Zentrum 10 - Universitätsklinikum Heidelberg

Ethik-Kommission I der Medizinischen Fakultät Heidelberg
Alte Glockengießerei 11/1
69115 Heidelberg

Zentrum 11 - Universitätsklinikum des Saarlandes

Ethik-Kommission der Ärztekammer des Saarlandes
Faktoreistr. 4
66111 Saarbrücken

Zentrum 12 - Universitätsklinikum Jena

Ethik-Kommission der Friedrich-Schiller-Universität Jena an der
Medizinischen Fakultät
Gebäude 1
Bachstr. 18
07740 Jena

Zentrum 13 - Universitätsklinikum Leipzig

Geschäftsstelle der Ethik-Kommission an der Medizinischen
Fakultät der Universität Leipzig
Haus: Karl-Sudhoff-Institut
Käthe-Kollwitz-Straße 82
04109 Leipzig

Zentrum 15 - Klinikum der Johannes-Gutenberg Universität Mainz

Ethik-Kommission bei der Landesärztekammer Rheinland-Pfalz
Deutschhausplatz 3
55116 Mainz

Zentrum 16 - Universitätsklinikum Mannheim

Med. Ethik-Kommission II der Medizinischen Fakultät Mannheim der Universität Heidelberg, Universitätsmedizin Mannheim
Maybachstr. 14
68169 Mannheim

Zentrum 17 - Klinikum der Universität München- Campus Großhadern

Ethikkommission der Med. Fakultät der LMU München
Pettenkoferstraße 8
80336 München

Zentrum 18 - Universitätsklinikum Regensburg

Ethikkommission der Medizinischen Fakultät der Universität Regensburg, Klinikum der Universität Regensburg
Franz-Josef-Strauß Allee 11
93053 Regensburg

Zentrum 19 - Universitätsklinikum Tübingen

Ethik-Kommission an der Medizinischen Fakultät der Eberhard-Karls-Universität und am Universitätsklinikum Tübingen
Gartenstr. 47
72074 Tübingen

Zentrum 20 - Universitätsklinikum Ulm

Ethikkommission der Universität Ulm
Helmholtzstr. 20
89081 Ulm

Zentrum 21 - Universitätsklinikum Würzburg

Ethik-Kommission bei der Medizinischen Fakultät der Universität Würzburg
Versbacher Str. 9
97078 Würzburg

Zentrum 22 - Universitätsklinikum Freiburg

Ethik-Kommission der Albert-Ludwigs-Universität Freiburg
Engelberger Str. 21
79106 Freiburg
